# The transcription factor Dach1 is essential for podocyte function

**DOI:** 10.1111/jcmm.13544

**Published:** 2018-03-02

**Authors:** Nicole Endlich, Felix Kliewe, Frances Kindt, Katharina Schmidt, Ahmed M. Kotb, Nadine Artelt, Maja T. Lindenmeyer, Clemens D. Cohen, Franziska Döring, Andreas W. Kuss, Kerstin Amann, Marcus J. Moeller, Nazanin Kabgani, Antje Blumenthal, Karlhans Endlich

**Affiliations:** ^1^ Department of Anatomy and Cell Biology University Medicine Greifswald Greifswald Germany; ^2^ Department of Anatomy and Histology Faculty of Veterinary Medicine Assiut University Assiut Egypt; ^3^ Nephrological Center Medical Clinic and Policlinic IV University of Munich Munich Germany; ^4^ Department of Functional Genomics University Medicine Greifswald Greifswald Germany; ^5^ Department of Nephropathology Institute of Pathology University Hospital Erlangen Erlangen Germany; ^6^ Department of Internal Medicine II, Nephrology and Clinical Immunology RWTH Aachen University Hospital Aachen Germany

**Keywords:** Dach1, dachd, differentiation, parietal epithelial cells, podocytes, transcription factors

## Abstract

Dedifferentiation and loss of podocytes are the major cause of chronic kidney disease. Dach1, a transcription factor that is essential for cell fate, was found in genome‐wide association studies to be associated with the glomerular filtration rate. We found that podocytes express high levels of Dach1 *in vivo* and to a much lower extent *in vitro*. Parietal epithelial cells (PECs) that are still under debate to be a type of progenitor cell for podocytes expressed Dach1 only at low levels. The transfection of PECs with a plasmid encoding for Dach1 induced the expression of synaptopodin, a podocyte‐specific protein, demonstrated by immunocytochemistry and Western blot. Furthermore, synaptopodin was located along actin fibres in a punctate pattern in Dach1‐expressing PECs comparable with differentiated podocytes. Moreover, dedifferentiating podocytes of isolated glomeruli showed a significant reduction in the expression of Dach1 together with synaptopodin after 9 days in cell culture. To study the role of Dach1 *in vivo*, we used the zebrafish larva as an animal model. Knockdown of the zebrafish ortholog Dachd by morpholino injection into fertilized eggs resulted in a severe renal phenotype. The glomeruli of the zebrafish larvae showed morphological changes of the glomerulus accompanied by down‐regulation of nephrin and leakage of the filtration barrier. Interestingly, glomeruli of biopsies from patients suffering from diabetic nephropathy showed also a significant reduction of Dach1 and synaptopodin in contrast to control biopsies. Taken together, Dach1 is a transcription factor that is important for podocyte differentiation and proper kidney function.

## 1 INTRODUCTION

Diabetes, hypertension and obesity are the major causes of chronic kidney disease (CKD) worldwide with rising tendency.^1^, ^2^ Approximately 5%‐10% of the entire population is affected by CKD, ending frequently in an end‐stage renal disease (ESRD) with the necessity for dialysis and kidney transplantation.^3^

Podocytes are frequently affected in CKD,^4^ whereby an effacement of foot processes and a detachment of single podocytes from the glomerular basement membrane (GBM) can be observed. As podocytes are postmitotic, injury and loss of podocytes can be compensated only partially by hypertrophy of the podocytes,^5^ resulting in the different forms of CKD like FSGS.^6^, ^7^ However, it is still under debate whether podocytes can proliferate as it was reported by Macconi and colleagues or whether PECs are able to differentiate into podocytes to some extent under specific circumstances.^8^, ^9^, ^10^, ^11^, ^12^, ^13^, ^14^, ^15^, ^16^

As podocyte loss and dedifferentiation are the major threats in CKD, we were interested to find out which proteins are involved in the dedifferentiation and differentiation process of podocytes. It was shown for the transdifferentiation of fibroblasts into several different cell types, for example neurons, that transcription factors play a crucial role in the regulation of such processes.^17^ Subsequently, different genome‐wide association studies pointed out that a single nucleotide polymorphism in the DACH1 locus is associated with an estimated glomerular filtration rate and CKD.^18^, ^19^, ^20^ Therefore, we focused on DACH1 for our analysis.

The *dachshund* gene (*dac*) that was originally discovered in the fruit fly *Drosophila*, where it is essential for normal eye and leg development, is highly conserved between human, mouse and zebrafish. Two homologues, *Dach1* and *Dach2*, are known in mice and humans^21^, ^22^ as well as the 4 homologues *dacha*,* dachb*,* dachc* and *dachd* in zebrafish.^23^, ^24^ *Dach1* ^−/−^ mice survive to birth, but die shortly thereafter by unknown reasons with no abnormalities in eye, limb and brain development.^25^ Also it was observed that in *mgb* ^−/−^ mice, a model system of congenital obstructive nephropathy and hydronephrosis, *Dach1* was one of the top up‐stream inhibited regulators.^26^

As *Dach1* is part of the *Eya‐Six‐Hox‐Pax* regulatory network, which has already been shown to be important for proper kidney development and function,^27^, ^28^, ^29^, ^30^, ^31^ we studied the influence of *Dach1* for the differentiation into podocytes in vivo and in vitro by cell culture experiments as well as the effects of a *dachd* knockdown (KD) in zebrafish larvae mediated *via* specific morpholinos (MO).

## 2 MATERIALS AND METHODS

### 2.1 Zebrafish stocks

The following zebrafish strains were used: *ET* and *CET*, previously described.^32^, ^33^ Zebrafish strains were breed in a pH‐ and temperature‐controlled facility as previously described.^34^ Zebrafish lines were grown and mated at 28.5°C, and eggs were directly collected in E3 media (5 mmol/L NaCl, 0.17 mmol/L KCl, 0.33 mmol/L CaCl_2_·2H_2_O, 0.32 mmol/L MgSO_4_·7H_2_O). For further experiments, eggs were incubated in a TB15 incubator (Thermo Fisher Scientific Inc., Waltham, MA USA) on 28.5°C and 100% humidity. E3 media was replaced daily. All experiments were performed in accordance with German animal protection law overseen by the “Landesamt für Landwirtschaft, Lebensmittelsicherheit und Fischerei, Rostock” of the federal state of Mecklenburg‐Western Pomerania.

### 2.2 Injection of morpholinos in fertilized zebrafish eggs

For injection of specific MO, the concentration of MO was set to 0.25, 0.5 or 1 mmol/L with injection solution (100 mmol/L KCl, 10 mmol/L Hepes) and phenol red solution (Sigma‐Aldrich, St. Louis, MO, USA). This mix was loaded to a Femtotip injection needle (Eppendorf AG, Hamburg, Germany, cat. no. 930000035) and connected to a Femtojet microinjector (Eppendorf AG) with pressure control. Freshly collected eggs were lined up on a 1% agarose gel, and the injection was carried out in the yolk of the eggs. *Dachd* vivo morpholino was injected in the cardial vene of larvae (3 dpf). The amount of injected MO was adapted by phenol red to minimize variability (2 nL). The MO were synthesized by Gene Tools LLC (http://www.gene-tools.com) and injected as described above. The sequence and identity of the different MOs can be viewed in Table S2.

### 2.3 Histology

For paraffin sections of mouse kidneys and human biopsies, samples were dehydrated and embedded into paraffin by standard procedures. Paraffin sections (5 μm) were performed on a Leica SM 2000R (Leica Microsystems, Wetzlar, Germany). After rehydration, sections were unmasked in citrate buffer (0.1 mol/L, pH 6.0) by heating for 5 minutes in a pressure cooker. The sections were stained with 1 mg/100 mL Hoechst 33342 (Sigma‐Aldrich) for 30 minutes. For the immunofluorescence double staining, samples were incubated with an antibody against synaptopodin (1:10; mouse; Progen Biotechnik GmbH, Heidelberg, Germany) and Dach1 (rabbit; Sigma‐Aldrich, HPA012672, 1:50) overnight. Samples were washed with 1× PBS for 3× 5 minutes and incubated with Cy3‐ and Cy2‐conjugated anti‐mouse/‐rabbit secondary antibodies (1:250; Jackson ImmunoResearch Laboratories, West Grove, PA, USA) for 1 hour. After additional washing, the samples were mounted in Mowiol (Carl Roth, Karlsruhe, Germany) for fluorescence microscopy.

Additionally, paraffin sections were stained using the Vectastain kit (Vector Laboratories, Burlingame, CA, USA) following manufacturer's instructions. Visualization was performed with DAB substrate kit (SK‐4100; Vector Laboratories) followed by nuclear staining with haematoxylin and mounting in Eukitt (Sigma‐Aldrich). In controls, PBS was used instead of primary antibody. Photographs were taken on an Olympus BX50 microscope equipped with an Olympus DP10 digital camera (Tokyo, Japan).

For fluorescence microscopy of zebrafish cryosections, larvae were prepared as previously described.^32^ Briefly, zebrafish larvae (3‐4 dpf) were fixed in 2% paraformaldehyde for 3 hours at room temperature, incubated in 30% sucrose at 4°C overnight and snap frozen in liquid nitrogen using Tissue‐Tek (Sakura, Staufen, Germany). Cryostat sections (60 μm) were stained with 1 mg/100 mL Hoechst 33342 (Sigma‐Aldrich) for 30 minutes. For nephrin staining, rabbit anti‐zebrafish nephrin antibody (Innovagen, Lund, Sweden) was incubated overnight at 4°C. After washing 3 times with 1× PBS, the Cy3‐conjugated anti‐rabbit secondary antibody (1:250; Jackson ImmunoResearch Laboratories) was applied for 30 minutes. The samples were washed and mounted in Mowiol for fluorescence microscopy.

### 2.4 Kidney biopsies

Kidney biopsies were archived at the Department of Nephropathology, Institute of Pathology, University Hospital Erlangen, Germany. The use of remnant kidney biopsy material was approved by the Ethics Committee of the Friedrich‐Alexander‐University of Erlangen‐Nürnberg, waiving the need for retrospective consent for the use of archived rest material (Re.‐No. 4415). Kidney biopsies of patients suffering from diabetic nephropathy have had the following parameter: age: 31‐73; average age: 53 (5 males and 2 female); proteinuria: 4‐14 g/d; diabetic mellitus type I and II, respectively. Control group: age: 44‐81; average age: 63 (8 males and 2 female). Sample fixation and preparation of the control and DN group were performed identically.

### 2.5 Cell culture

PECs were cultivated in EBM medium (Lonza Group Ltd., Basel, Switzerland) supplemented with EGM‐MV singlequots T75 (Lonza Group Ltd.) as reported.^35^ PECs were passaged every 3‐4 days. Transfection of PECs was performed with 1 μg/mL GFP‐ and the Dach1‐GFP plasmids (OriGene Technologies, Rockville, MD, USA) by lipofectamine 2000 transfection (Invitrogen, Carlsbad, CA, USA) in serum‐free RPMI medium (Lonza Group Ltd.) according to the manufacturer's instructions. Cells were used 48 hours after transfection for immuncytochemistry, for protein and RNA isolation. Immortalized murine podocytes were cultured in serum‐containing RPMI medium as described.^36^ Podocytes were used after differentiation for at least 6 days at 38°C.

### 2.6 Immunocytochemistry

PECs were fixed with 2% paraformaldehyde for 10 minutes, permeabilized by 0.3% Triton‐X (Sigma‐Aldrich) for 3 minutes and blocked for 1 hour with blocking solution (2% FBS, 2% BSA and 0.2% fish gelatine in PBS). Primary antibodies were diluted in blocking solution and incubated for 1 hour on cells: anti‐synaptopodin (mouse, Progen, 61094, 1:100) and anti‐Dach1 (rabbit, Sigma‐Aldrich, HPA012672, 1:100). Secondary antibodies were diluted in blocking solution and incubated with the cells for 30 minutes: anti‐mouse‐Cy3 (Jackson ImmunoResearch Laboratories, 1:300) and anti‐rabbit‐Cy3 (Jackson Immuno Research, 1:300). For the visualization of the actin cytoskeleton, cells were stained with Alexa Fluor‐546 phalloidin (1:100; Thermo Fisher Scientific, Waltham, MA, USA) for 30 minutes. For nuclei staining, DAPI (1:150; Sigma‐Aldrich) was used for 5 minutes. All samples were mounted in Mowiol (Carl Roth, Karlsruhe, Germany) and used for laser scanning microscopy (LSM) or structured illumination microscopy (SIM).

### 2.7 Laser scanning microscopy and structured illumination microscopy

Images were captured either with a Leica TCS SP5 confocal microscope (Leica Microsystems), 40×/63× oil immersion objectives in the Leica Application Suite software (Leica Microsystems, Version 2.6.0) or with a structured illumination microscope (SIM; ELYRA, Carl Zeiss Microscopy GmbH, Jena, Germany), 63× oil immersion objective.

### 2.8 Electron microscopy

Zebrafish larvae (5 dpf) were fixed in 4% glutaraldehyde and 1% paraformaldehyde in 0.1 mol/L HEPES‐buffer containing 0.1% MgCl_2_ and 0.5% CaCl_2_ plus 1% sucrose at 4°C overnight. Fixed larvae were washed 3× 30 minutes with PBS, postfixed in 2% osmium tetroxide for 2 hours and dehydrated through a graded ethanol series. After propylenoxide wash (2× 15 minutes), probes were embedded in EPON 812 (SERVA, Heidelberg, Germany). Sections were cut on an Ultracut UCT ultramicrotom (Leica Microsystems) and contrasted with 5% uranyl acetate and lead citrate. All grids were examined with a LIBRA^®^ 120 transmission electron microscope (Carl Zeiss).

### 2.9 Calculations

For calculation of the synaptopodin‐positive cell ratio, GFP‐positive cells from 5 different biological replicates (PEC‐Ctrl and PEC‐Dach1), each with >150 cells, were counted for the absence or presence of synaptopodin using the LSM.

To identify synaptopodin‐positive cells expressing GFP or not, cells were analysed by SIM (ELYRA, Zeiss). Only cells with a strong expression of synaptopodin were counted in a defined area in PEC‐Ctrl and PEC‐Dach1 (3 independent experiments). Calculations were carried out in Prism, and statistical analysis was performed using Student's *t* test.

### 2.10 RNA extraction, cDNA synthesis, qRT‐PCR and RNA sequencing

Samples from cells or tissues were dissolved in 1 mL Tri‐Reagent (Sigma‐Aldrich) according to manufacturer's instructions. For cDNA synthesis, 1 μg of the isolated total RNA was transcribed using the QuantiTect Reverse Transcription Kit (Qiagen, Hilden, Germany). The quantitative real‐time PCR (qRT‐PCR) analysis was performed on a LightCycler Nano (Roche Diagnostics GmbH, Mannheim, Germany) using the iTaq Universal SYBR Green Supermix (Bio‐Rad, Hercules, CA, USA) with *Actb* and *Gapdh* as reference genes. Relative quantifications of the mRNA levels were performed by the efficiency corrected calculation model by Pfaffl^37^ and are shown with standard deviations from 3 biological replicates. Primers used for mouse samples can be viewed in Table S1.

Expression levels of mRNA corresponding to *Dach1* (ENSMUSG00000055639) and synaptopodin (*Synpo*, ENSMUSG00000043079) were also determined by RNA sequencing of samples isolated from murine glomeruli. Sequencing was carried out on a SOLiD 5500xl sequencing platform (Life Technologies, Carlsbad, CA, USA) as previously described.^38^ Sequence analysis was based on GRCm38/mm10. Benjamini‐Hochberg adjusted Wald test *P*‐values (*P*_adj_) were determined to identify significant differences in gene expression between samples.

### 2.11 Patients and microarray analysis

Human renal biopsy specimens and Affymetrix microarray expression data were procured within the framework of the European Renal cDNA Bank—Kröner‐Fresenius Biopsy Bank.^39^, ^40^ Biopsies were obtained from patients after informed consent and with approval of the local ethics committees. Following renal biopsy, the tissue was transferred to RNase inhibitor and microdissected into glomeruli and tubulointerstitium. Total RNA was isolated from microdissected glomeruli, reverse transcribed and linearly amplified according to a protocol previously reported.^41^ The microarray expression data used in this study came from individual patients with DN. Pre‐transplantation kidney biopsies from living donors (LD) were used as control renal tissue. Fragmentation, hybridization, staining and imaging were performed according to the Affymetrix Expression Analysis Technical Manual (Affymetrix, Santa Clara, CA, USA). CEL file normalization was performed with the Robust Multichip Average method using RMAExpress (Version 1.0.5) and the human Entrez‐Gene custom CDF annotation from Brain Array version 18 (http://brainarray.mbni.med.umich.edu/Brainarray/default.asp). To identify differentially expressed genes, the SAM (Significance Analysis of Microarrays) method was applied using TiGR (MeV, Version 4.8.1).^42^ A *q*‐value below .05 was considered to be statistically significant.

### 2.12 Isolation of mouse kidneys and glomeruli by magnetic dynabeads

Glomeruli of nephrin:CFP mice were isolated with magnetic dynabeads as previously described.^43^ Afterwards, isolated glomeruli were cultured on collagen IV‐coated μ‐slides (ibidi GmbH, Munich, Germany) in phenol red‐free RPMI 1640 medium (Lonza Group Ltd.) supplemented with 10% FBS (Invitrogen), 100 U/mL penicillin and 0.1 mg/mL streptomycin (Life technologies). After 0 and 9 days, RNA samples were prepared.

### 2.13 Protein isolation

Protein isolates of transgenic PECs, podocytes, mice glomeruli and kidneys were performed by lysis of the samples in Pierce IP lysis buffer (Thermo Fisher Scientific Inc.) supplemented with Halt Protease Inhibitor (Thermo Fisher Scientific Inc.). Lysis was performed for 20 minutes by shaking intensively at 4°C in a Thermomixer Comfort (Eppendorf AG). For zebrafish larvae, homogenization and lysis was carried out using 2 mL lysing tubes with 1.4 mm ceramic beads (MP Biomedical, Santa Ana, CA, USA) in a FastPrep‐24 homogenizer (20 seconds at 4 m/s; MP Biomedicals) prior to shaking for 20 minutes at 4°C. Furthermore, lysed samples were centrifuged at 15 000 ***g*** for 20 minutes. Supernatant (protein isolates) was stored at −20°C.

### 2.14 Western blot analysis

For Western blot analysis, protein homogenates from transgenic PECs, podocytes and mouse glomeruli or kidneys were quantified using the Pierce BCA protein assay kit (Thermo Fisher Scientific). After addition of SDS‐PAGE sample buffer (0.35 mol/L Tris pH 6.8, 0.35 mol/L SDS, 30% (v/v) glycerol, 10 mmol/L β‐mercaptoethanol, 0.175 mmol/L bromophenol blue), the cell lysates were boiled at 95°C for 5 minutes and separated on a 4‐20% gradient Mini‐Protean TGX Gel (Bio‐Rad). Furthermore, separated proteins were blotted on nitrocellulose membranes using the Trans‐Blot Turbo RTA Transfer Kit (Bio‐Rad) and the Trans‐Blot Turbo Transfer System (Bio‐Rad) at 2.5 A/25 V for 5 minutes. Blots were washed in 1× TBST (50 mmol/L Tris, 150 mmol/L NaCl, 10 mmol/L CaCl_2_, 1 mmol/L MgCl_2_ supplemented with Tween‐20 0.1%; AppliChem) and blocked with 5% milk powder for 1 hour at room temperature. Moreover, primary antibodies were diluted in 2.5% milk powder (in 1× TBST) and incubated with the blots overnight at 4°C. After intensive washing in 1× TBST, blots were incubated with secondary antibodies for 60 minutes, washed again, developed with the ECL Prime Western Blotting Detection Reagent (GE Healthcare, Little Chalfont, UK) and visualized on X‐ray films (GE Healthcare). For normalization and usage of alternative antibodies on the same blot, blots were stripped. Antibodies were used at the following final concentrations: anti‐synaptopodin (1:500; sc‐21537; Santa Cruz Biotechnology, Santa Cruz, CA, USA), anti‐Dach1 (1:1,500; HPA012672; Sigma‐Aldrich), anti‐WT1 (1:800; sc‐192; Santa Cruz Biotechnology), anti‐Pax2 (1:1,500; ab38738; Abcam, Cambridge, UK) and anti‐GAPDH (1:5,000; sc‐25778; Santa Cruz Biotechnology). For the relative quantification, developed X‐ray films were scanned and analysed using ImageJ (version 1.49 m; NIH, Bethesda, MD, USA).

### 2.15 Statistical analysis

**Figure 1:**
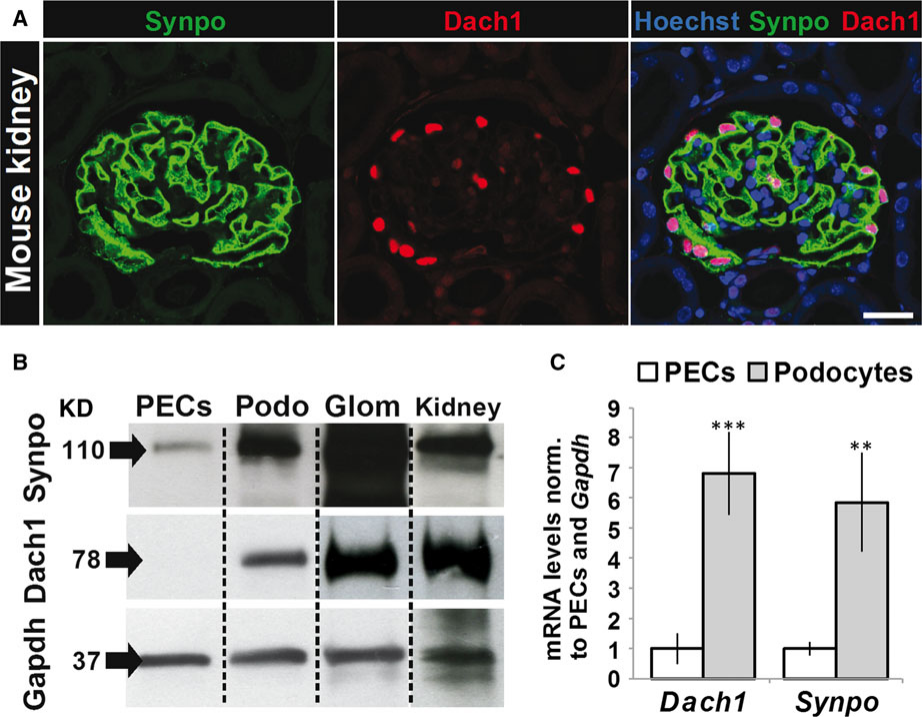
Dach1 and synaptopodin expression in mouse kidney and cell culture. A, Expression of Dach1 and synaptopodin in mouse glomeruli imaged by laser scanning microscopy (n = 3). Mouse kidney paraffin sections were stained with Hoechst (DNA, blue) and with antibodies against synaptopodin (green) and Dach1 (red). Scale bar represents 25 μm. B, Dach1 and synaptopodin protein expression were compared between PECs, podocytes (Podo), isolated glomeruli (Glom) and mouse kidney by Western blots (n = 3). Gapdh was used as a reference protein. C, Relative mRNA levels of *Dach1* and *Synpo* in PECs (white bars, means ± SD, n = 5) and podocytes (grey bars, means ± SD, n = 5) were determined by qRT‐PCR. Normalization was performed against PEC expression levels and *Gapdh* as reference gene. ***P <* *.001, **P <* *.01

**Figure 2:**
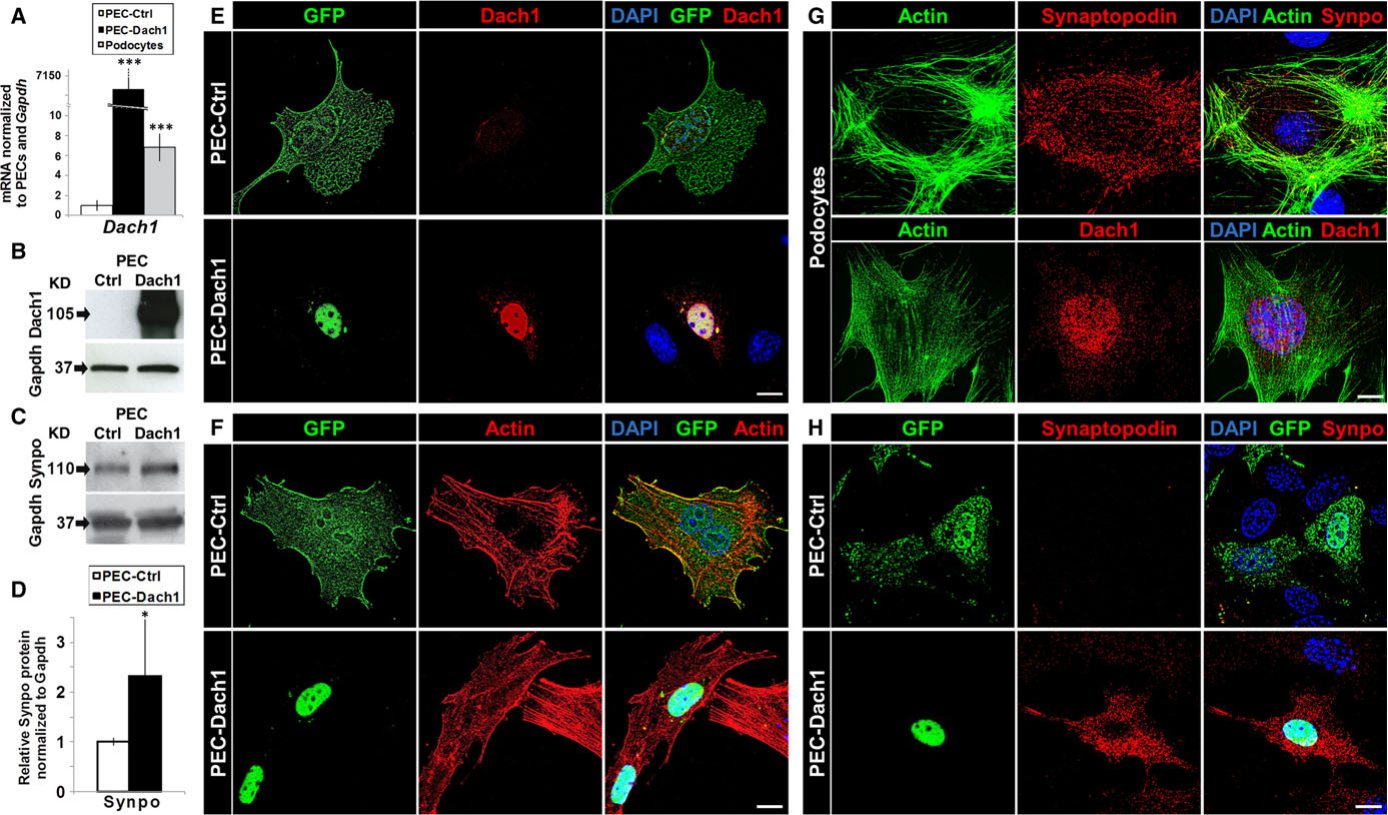
Expression of Dach1 in PECs induced an up‐regulation of the podocyte‐specific protein synaptopodin. A, Transfection of PECs with pDach1‐GFP (PEC‐Dach1: black bar, mean ± SD, n = 5) causes a strong Dach1 expression compared to cells transfected with pGFP (PEC‐Ctrl: white bar, mean ± SD, n = 5). In addition, Dach1 is up‐regulated to a comparable level as observed in podocyte cell culture (grey bar, mean ± SD, n = 5). B, Dach1 protein up‐regulation in PEC‐Dach1 was shown by Western blot analysis. C, Synaptopodin protein up‐regulation in PEC‐Dach1 was shown by Western blot analysis. D, Relative synaptopodin protein levels on Western blots were quantified for PEC‐Ctrl (white bar, mean ± SD, n = 5) and PEC‐Dach1 (black bar, mean ± SD, n = 5) and revealed an up‐regulation of synaptopodin protein for PEC‐Dach1. E‐H, Shown are either PECs (E, F, H) or podocytes (G). The up‐regulation of Dach1 (E) and synaptopodin (H) in PEC‐Dach1 was shown by immunofluorescence. F, Phalloidin staining was performed to analyse possible effects on the actin cytoskeleton, whereby no effects could be observed. Scale bars represent 10 μm (E‐H). Colours are green for GFP or Dach1‐GFP expression (E, F, H) or the actin‐cytoskeleton (G), red for either Dach1 (E, G), the actin cytoskeleton (F) or synaptopodin (G, H) and blue for DAPI‐stained DNA (E‐H). ***P <* *.001, **P <* *.01, *P <* *.05

**Figure 3:**
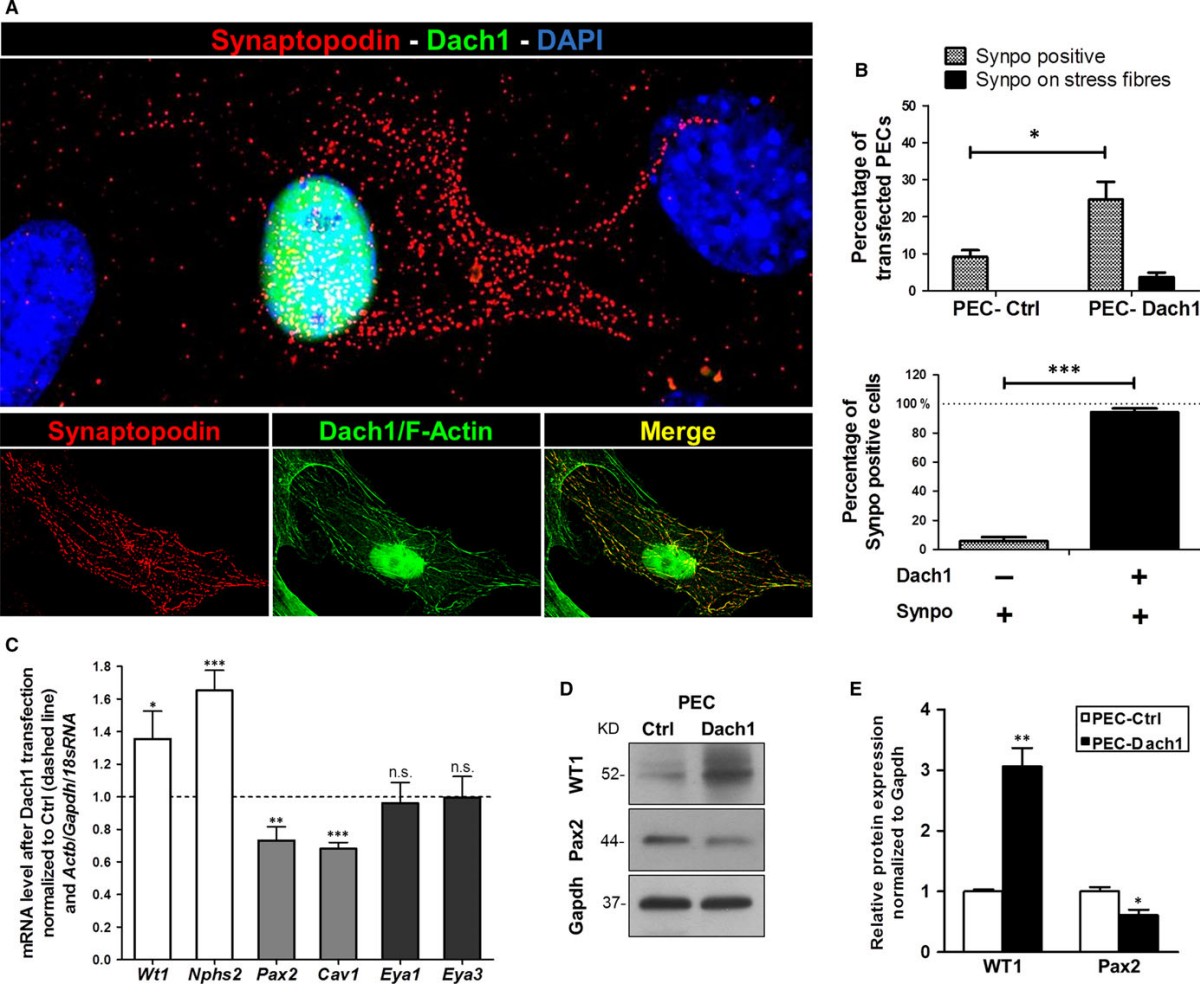
Effect of Dach1 expression in PECs on synaptopodin and further PEC/podocyte proteins. A, The up‐regulation of synaptopodin (red) and co‐localization with F‐actin (green) was shown by immunofluorescence and super resolution microscopy in PEC‐Dach1. B, Synaptopodin‐positive cells are significantly increased in PEC‐Dach1 as compared to PEC‐Ctrl (upper diagram, n = 5 experiments with >100 cells per experiment). Synaptopodin‐positive PECs were almost always positive for Dach1 expression (lower diagram, n = 5 experiments with >100 cells). C, Podocyte marker genes (*Wt1*,* Nphs2*), PEC marker genes (*Pax2*,* Cav1*) and differentiation/development regulators (*Eya1, Eya3*) were quantified by qRT‐PCR analysis of total mRNA isolated from PEC‐Ctrl and PEC‐Dach1. qRT‐PCR experiments were normalized to PEC‐Ctrl (dashed line). *Actin*,* Gapdh* and *18sRNA* served as reference genes (n ≥ 3, means ± SEM). D, WT1 up‐regulation and Pax2 down‐regulation in PEC‐Dach1 were shown by Western blot analysis. E, Relative Wt1 and Pax2 protein levels on Western blots were quantified for PEC‐Ctrl (white bars) and PEC‐Dach1 (black bars) and revealed an up‐regulation of WT1 (n = 4, means ± SEM) and a down‐regulation of Pax2 (n = 6, means ± SEM) in PEC‐Dach1. Western blots were normalized to PEC‐Ctrl and Gapdh levels. ***P <* *.001, **P <* *.01, *P <* *.05

Error bars represent standard deviations (SD) in Figures 1, 2 and 6 and standard error of the mean (SEM) in Figures 3 and 5 among replicate experiments. Statistical significance of differences between data sets was analysed using the unpaired Student's *t* test and expressed as *P*‐values.

## 3 RESULTS

### 3.1 Dach1 is specifically expressed in podocytes in vivo and in vitro

To study the localization of Dach1, paraffin sections of mouse kidneys were stained with an antibody specific for Dach1 and analysed by LSM. As shown in Figure 1A, Dach1 is mainly expressed in the glomerulus. Co‐localization studies with the podocyte‐specific protein synaptopodin revealed that Dach1 is strongly expressed in the nuclei of podocytes (Figure 1A). Western blot analysis in mice showed much stronger signals for synaptopodin and Dach1 of isolated glomeruli and kidney samples compared to cultured mouse podocytes and PECs (Figure 1B). Additionally, cultured podocytes expressed significantly more mRNA of *Synpo* (585 ± 166%) and *Dach1* (682 ± 138%) compared to cultured PECs (Figure 1C), suggesting a correlation between expression of Dach1 and synaptopodin.

### 3.2 Expression of Dach1 in PECs induces an up‐regulation of synaptopodin

To study whether the cell determination factor Dach1 is able to convert PECs into podocyte‐like cells, we transfected PECs with a plasmid encoding for Dach1 coupled to GFP (PEC‐Dach1). As a control, PECs were transfected with a plasmid encoding for GFP only (PEC‐Ctrl). The transfection efficiency was quantified by qRT‐PCR and Western blot (Figure 2A,B). To examine whether PECs become podocyte‐like cells, we looked for the expression of synaptopodin, a specific marker for differentiated podocytes in vivo and in vitro. We found by Western blot that the expression of synaptopodin was increased to 246 ± 95% after the transfection of PECs with a plasmid encoding for Dach1 (Figure 2C,D).

### 3.3 Synaptopodin is localized in dense bodies along actin fibres in PEC‐Dach1

As synaptopodin is an actin‐binding protein, we studied the localization of synaptopodin in PEC‐Dach1 compared to differentiated podocytes by SIM. Using an antibody specific for Dach1, PEC‐Ctrl showed a faint staining for Dach1 in contrast to PEC‐Dach1 (Figure 2E) and podocytes (Figure 2G). Expression of Dach1‐GFP, which was localized almost exclusively in the nucleus (Figure 2E,F,H), had no influence on the cell morphology and the actin cytoskeleton of PECs as shown in Figure 2F. However, after the expression of Dach1 in PECs, synaptopodin was strongly up‐regulated in PEC‐Dach1 in contrast to PEC‐Ctrl (Figure 2H). Moreover, synaptopodin was often distributed in a punctate pattern as shown in Figures 2H and 3A, according to its well‐known localization in dense bodies along actin fibres.

For quantification of synaptopodin‐positive cells, more than a hundred PEC‐Dach1 and PEC‐Ctrl cells were analysed by SIM. We found an increase of synaptopodin‐positive cells from 9.2 ± 1.8% in PEC‐Ctrl to 24.7 ± 4.8% in PEC‐Dach1. Interestingly, the number of synaptopodin‐positive cells that expressed synaptopodin strongly along actin fibres increased from 0% in PEC‐Ctrl to 3.8 ± 1.2% in PEC‐Dach1 (Figure 3B, upper graph). Notably, 94.3 ± 2.7% of the synaptopodin‐positive PECs were also Dach1‐positive, and only 5.7 ± 2.7% of the synaptopodin‐positive PECs were Dach1‐negative (Figure 3B, lower graph).

Summarizing, the expression of Dach1 in PECs induces an up‐regulation of the podocyte‐specific protein synaptopodin that is localized in dense bodies along actin fibres.

### 3.4 Dach1 and the expression of various podocyte and PEC markers

As Dach1 regulates the expression of the podocyte‐specific differentiation marker synaptopodin, the expression of other podocyte‐ and PEC‐specific markers was analysed by qRT‐PCR in PEC‐Ctrl and PEC‐Dach1. For the following selected podocyte markers, we found a significant increase of the ratio normalized to PEC‐Ctrl: *Wt1* 136 ± 17% and podocin (*Nphs2*) 165 ± 12% (Figure 3C). In contrast, the expressions of the PEC markers *Pax2* and caveolin‐1 (*Cav1*) were decreased significantly to 73 ± 8% and 68 ± 4%, respectively. Additionally, we studied the expression of *Eya1* and *Eya3*. Both genes are known to be general regulators in differentiation/dedifferentiation processes. Our analysis revealed that expression of *Eya1* and *Eya3* was unaffected by Dach1 expression (96 ± 13% and 99 ± 13%, respectively) (Figure 3C). Western blots demonstrated a significant increase of WT1 by 206 ± 31% and a reduction in Pax2 by 39 ± 9% after transfection with Dach1 (Figure 3D,E).

### 3.5 Decreased Dach1 expression in cultured glomeruli correlates with reduced synaptopodin levels

**Figure 4:**
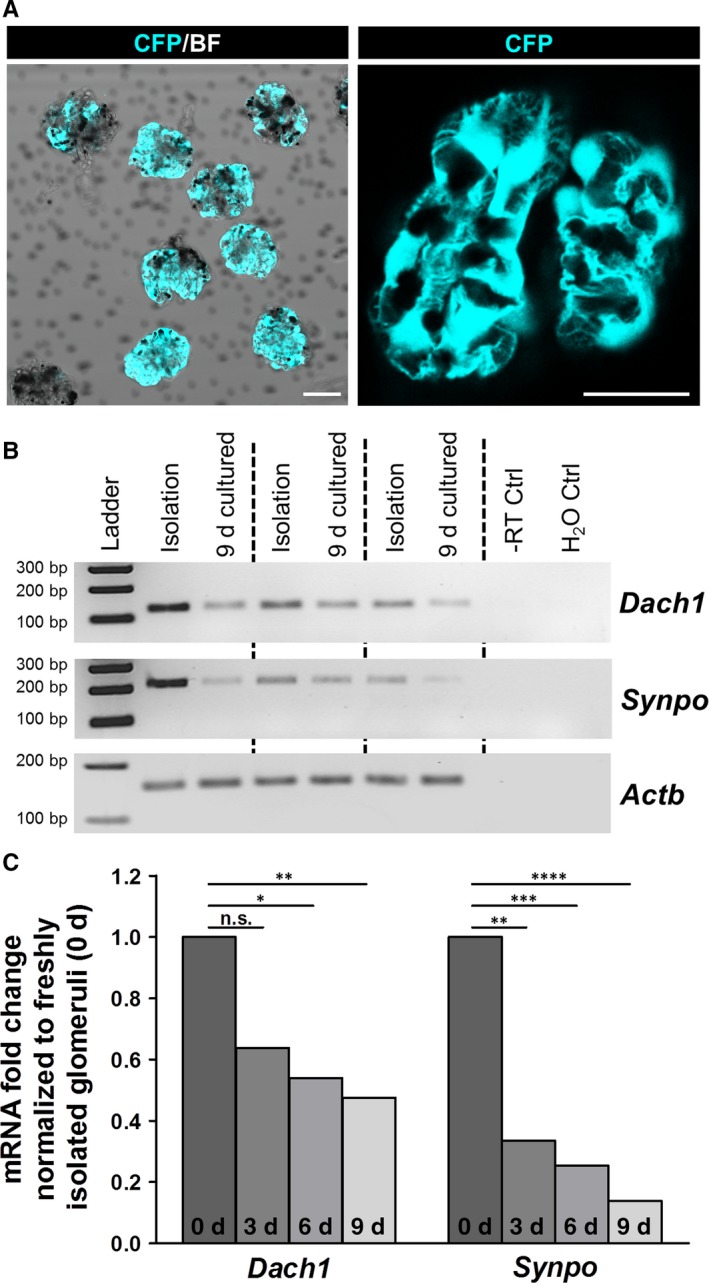
*Dach1* and *Synpo* mRNA levels were reduced after cultivation of isolated glomeruli. A, Isolated glomeruli express CFP in podocytes under control of the nephrin promoter. BF—bright field. Scale bars represent 25 μm. B, As determined by RT‐PCR,* Dach1* and *Synpo* mRNA expression significantly decreased after cultivation of glomeruli for 9 days (n = 3). C, RNA sequencing of isolated glomeruli from day 0, 3, 6 and 9 confirmed a significant decrease in the mRNA of *Dach1* and *Synpo* during dedifferentiation. ****P <* *.0001, ***P <* *.001, **P <* *.01, *P <* *.05

Further we investigated the expression of Dach1 during the spontaneous dedifferentiation of podocytes in culture.^43^ To this end, glomeruli of nephrin:CFP mice were isolated (Figure 4A), cultured for 9 days and RNA samples were prepared. As determined by RT‐PCR, mRNA expression of *Dach1* and *Synpo* was decreased after 9 days in culture, whereas β‐actin (*Actb*) levels remained unchanged (Figure 4B). Moreover, RNA sequencing of glomeruli confirmed a significant reduction in *Dach1* (fold change: 0.47; *P*_*adj*_ = .0031) and *Synpo* (fold change: 0.14; *P*_*adj*_ = 4.3 × 10^−15^) transcripts after 9 days (Figure 4C). In contrast to that, other podocyte markers such as *Cd2ap* (fold change 1.87; *P*_*adj*_ = .0026) and podoplanin (*Pdpn*; fold change 3.07; *P*_*adj*_ = .026) were even significantly up‐regulated.

### 3.6 Knockdown of Dachd in zebrafish larvae compromises the glomerular filtration barrier

**Figure 5:**
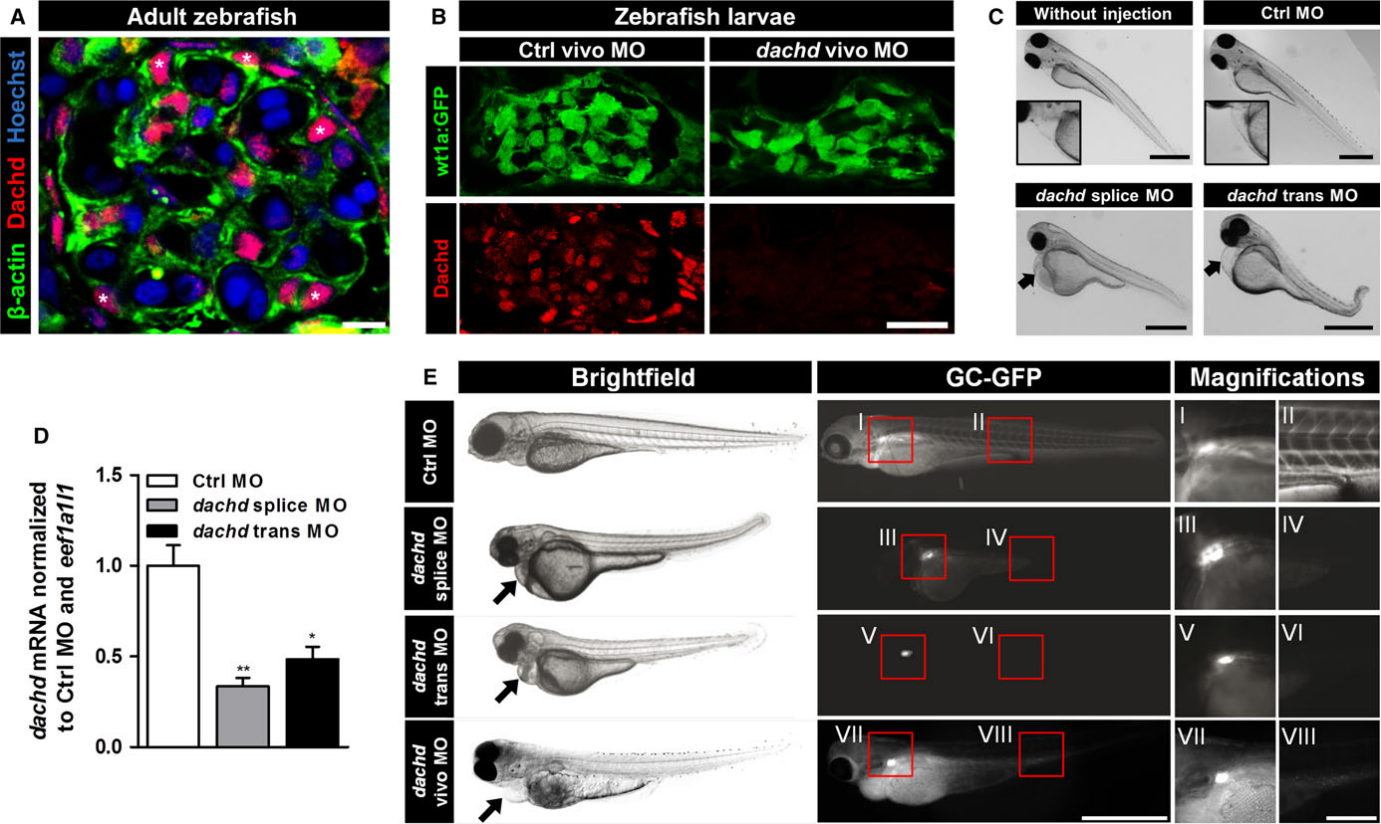
Knockdown (KD) of Dachd in zebrafish larvae compromised the glomerular filtration barrier. A, Expression of Dachd and β‐actin in mesonephric glomeruli of the adult zebrafish imaged by laser scanning microscopy. Paraffin sections were stained with Hoechst (DNA, blue) and with antibodies against β‐actin (green) and dachd (red). Scale bar represents 5 μm. B, Cryosections of 5 dpf wt1a:gfp zebrafish larvae showed a strong down‐regulation of Dachd expression after injection of *dachd* vivo morpholinos (MO). Scale bar represents 20 μm. C, Zebrafish eggs were injected with either control MO (Ctrl MO), *dachd* splice or trans MO. Brightfield images of zebrafish larvae (3 dpf) showed oedema formation after *dachd* splice or trans MO injection (arrows in C; n = 3). Scale bars represent 250 μm. D, The *dachd* mRNA was determined by qRT‐PCR analysis. Total mRNA extracts of whole larvae (3 dpf) injected with Ctrl MO (white bar), *dachd* splice (grey bar) or trans MO (black bar) (mean ± SEM, n = 5, 25 larvae per group) were used for cDNA synthesis. Data sets were normalized to Ctrl MO‐injected samples and the reference gene *eef1a1l1*. **P < .01, *P < .05. E, Studying the function of the glomerular filtration barrier by analysis of the GFP signals in *CET* larvae (wt1a:GFP x l‐fabp:GC‐GFP) in Ctrl, *dachd* splice and *dachd* trans MO‐injected larvae (3 dpf; n = 5) and *dachd* vivo MO‐injected larvae (6 dpf; n = 5). The fluorescence in the blood was reduced after the Dachd KD as a result of a leaky filtration barrier. In *dachd* splice, trans and vivo MO‐injected larvae only the green fluorescence of GFP‐positive podocytes was visible (I, III, V, VII: GFP‐expressing podocytes; II: GC‐GFP in blood vessels; IV, VI, VIII: leakage of the filtration barrier resulted in the loss of GC‐GFP). The *dachd* splice MO affects the correct splicing of the exon 2. The *dachd* trans and vivo MO binds in exon 1 of the *dachd* transcripts. Scale bars represent 250 μm, respectively, 50 μm (magnifications)

As Dach1 induces the expression of the podocyte‐specific protein synaptopodin, we wanted to study the role of Dach1 for renal function in a living organism. As the zebrafish had been shown to be an ideal model to study kidney function,^32^, ^33^, ^44^, ^45^, ^46^, ^47^, ^48^, ^49^ we induced a KD of the Dach1 ortholog, Dachd*,* by the injection of 3 specific MOs—splice blocking MO (*dachd* splice MO), translational blocking MO (*dachd* trans MO) into fertilized zebrafish eggs and Vivo‐MO (*dachd* vivo MO) into the cardial vene of 3 dpf larvae. In the mesonephric as well as in the pronephric glomerulus, Dachd localized to the nuclei of podocytes (asterisks in Figure 5A and left panel of Figure 5B). The KD efficiency of MOs was verified by immunofluorescence staining and qRT‐PCR (Figure 5B,D and Figure S2). Three days postfertilization (dpf), 51 ± 20% of the dachd splice and 48 ± 25% of the dachd trans injected zebrafish larvae developed pericardial and yolk sac oedema, a hallmark of glomerular failure (n = 10 with at least 50 injected fertilized zebrafish eggs). In contrast, the control MO (Ctrl MO) injected larvae showed no difference compared to untreated larvae (Figure 5C). To study the filtration barrier in living zebrafish larvae, we used the specific zebrafish strain *CET* that expresses GFP in podocytes (Figure 5E I, III, V, VII) and the vitamin‐binding protein D coupled to GFP (GC‐GFP) in the blood (Figure 5E II, IV, VI, VIII), which is too big to pass the intact filtration barrier as we have described earlier.^33^ After KD of Dachd in zebrafish larvae, we observed a decrease in the intravascular fluorescence intensity (Figure 5E IV, VI, VIII) in contrast to Ctrl MO larvae (Figure 5E II) indicating a leaky filtration barrier.

**Figure 6:**
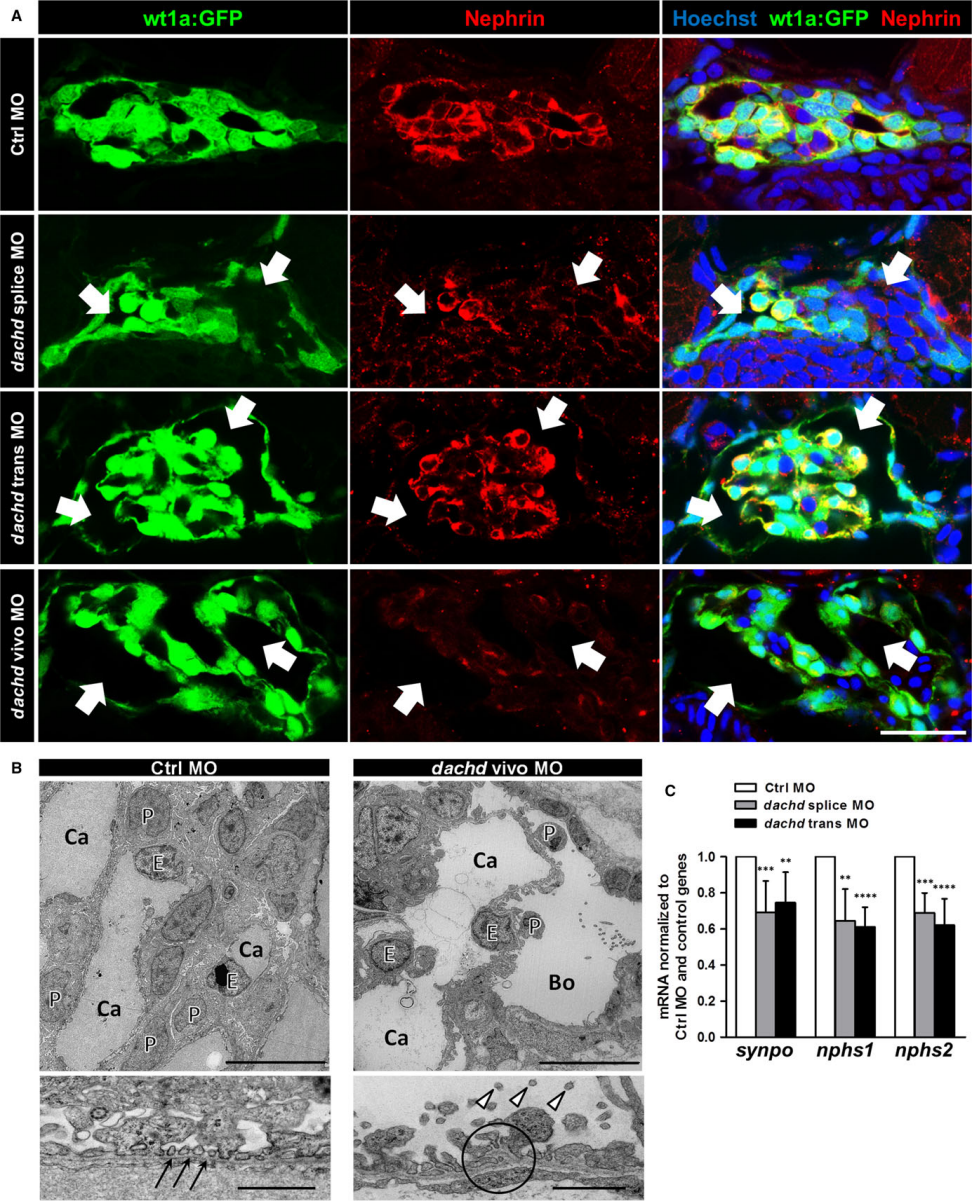
Knockdown (KD) of Dachd in zebrafish larvae affected podocyte gene expression and induced foot process effacement. A, Cryosections of 3 dpf zebrafish larvae showed an increase in Bowman's space (arrows) after the KD of Dachd (n = 3). Nephrin staining is shown in the middle panel. Scale bar represents 20 μm. B, Electron microscopy of Dachd KD and Ctrl morpholinos (MO) zebrafish larvae. Ctrl MO larvae showed a normally structured filtration barrier with intact foot processes (arrows). Foot processes of *dachd* vivo MO larvae were disordered with microvilli‐like structures (arrowheads) protruding in an increased Bowman's space (Bo). glomerular basement membrane and endothelial cells were also affected by MO treatment (circle). Scale bars represent 10 μm in the upper and 1 μm in the lower pictures. P, podocyte; E, endothelial cell; Ca, capillary. C, The expression of podocyte genes (*synpo*,* nphs1* and *nphs2*) was determined by qRT‐PCR analysis. Total mRNA extracts of whole larvae (3 dpf) injected with Ctrl MO (white bars, means ± SD, n = 5), *dachd* splice MO (gray bars, means ± SD, n = 5) or *dachd* trans MO (black bars, means ± SD, n = 5) were used for cDNA synthesis. Data sets were normalized to Ctrl MO‐injected samples and the reference genes *rpl13*,* eef1a1l1* and *18sRNA*. ****P <* *.0001, ***P <* *.001, **P <* *.01, *P <* *.05

Immunohistological analysis showed green fluorescent podocytes indicating that the expression of wt1a was not affected by the Dachd KD (Figure 6A). However, we observed an enlarged Bowman's space and a significant reduction in the nephrin staining of podocytes in response to the Dachd KD (Figure 6A).

To exclude glomerular failure caused by developmental effects, we used *dachd* vivo MO that were injected into 5 dpf zebrafish larvae. We found that *dachd* vivo MOs also induced a leakage of the glomerular filter barrier (Figure 5E) and a reduction in the nephrin expression (Figure 6A) indicating that Dachd is essential for proper blood filtration and function of the fully developed pronephros.

### 3.7 Knockdown of Dachd induces podocyte foot process effacement and decreases the expression of specific podocyte proteins in zebrafish larvae

Electron microscopy of the *dachd* vivo MO larvae showed an enlarged Bowman's space and an effacement of podocyte foot processes (Figure 6B). Furthermore, podocytes developed microvilli‐like structures protruding into the urinary space (arrow heads in Figure 6B). Moreover, we observed an irregular morphology of the GBM in some regions (encircled area in Figure 6B). In contrast, the Ctrl MO larvae revealed a normal glomerular structure with normally developed foot processes (arrows in Figure 6B). To find out whether these morphological changes are associated with the down‐regulation of the mRNA of podocyte‐specific proteins, we determined the expression of synaptopodin (s*ynpo*), nephrin (*nphs1*) and podocin (*nphs2*) mRNAs by qRT‐PCR (Figure 6C). We observed a significant down‐regulation of the mRNA of *synpo* by 31 ± 18% and 25 ± 17%, of *nphs1* by 35 ± 18% and 39 ± 11% and of *nphs2* by 31 ± 11% and 38 ± 15% in dachd splice and trans MO zebrafish larvae, respectively, in contrast to Ctrl MO larvae (Figure 6C). This suggests a regulatory role of Dachd for podocyte‐specific proteins in zebrafish larvae.

### 3.8 Patients with diabetic nephropathy show reduced DACH1 and synaptopodin expression in glomeruli

**Figure 7:**
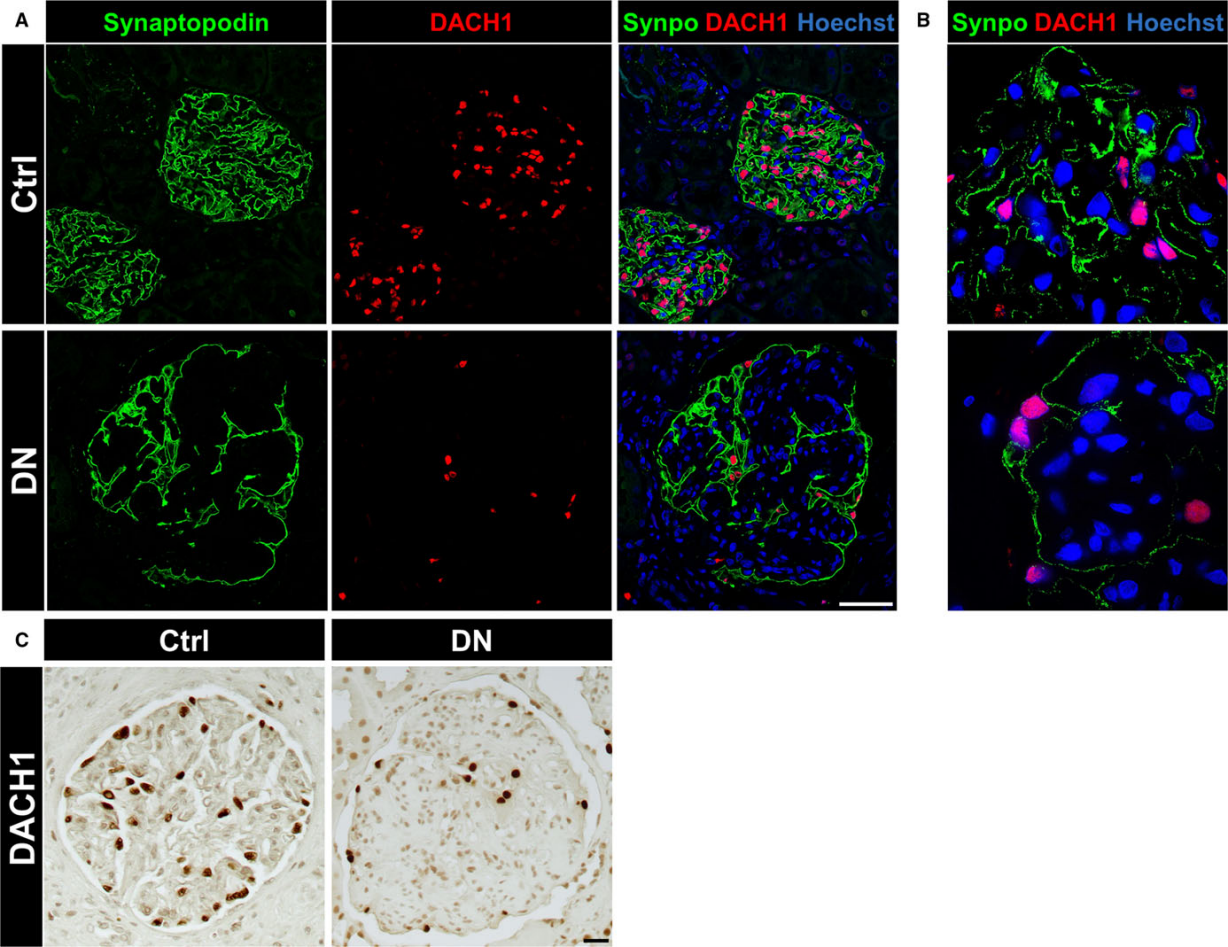
Diabetic nephropathy in human patients caused a severe down‐regulation of DACH1 and synaptopodin. A, The expression of DACH1 (red) and synaptopodin (green) was reduced in glomeruli of patients suffering from diabetic nephropathy (DN) as compared to control tissue (Ctrl) imaged by laser scanning microscopy (each n = 3). Scale bar represents 50 μm. B, Expression of DACH1 and synaptopodin in human kidneys imaged by super resolution microscopy (structured illumination microscopy—SIM). The expression of DACH1 and synaptopodin was reduced in DN patients in contrast to the controls. C, Immunohistochemistry of human kidneys showed the reduced DACH1 expression in DN compared to controls. Scale bar represents 20 μm

To analyse the regulation of DACH1 in diabetic nephropathy (DN), we assessed the mRNA expression level of DACH1 in microdissected glomeruli from renal biopsies of patients suffering from DN (n = 7) and compared them with healthy LD (n = 18). Patients with DN displayed a significant decrease in DACH1 expression compared to controls (fold change: 0.582; *q *=* *.00074) that is in agreement with the staining of human biopsies (Figure 7C).

To find out whether the expression of DACH1 is associated with the expression of synaptopodin, we stained biopsies from patients with DN. The control biopsies showed a strong expression of DACH1 in the nuclei of podocytes and an intensive staining of synaptopodin along the foot processes of podocytes (Figure 7A,B). However, the pictures that were taken from biopsies of patients suffering from DN under the same conditions revealed a significant reduction of DACH1 and synaptopodin, as detected by LSM and SIM (Figure 7A,B).

## 4 DISCUSSION

Dedifferentiation and loss of podocytes is a major cause for the development of severe kidney diseases like FSGS and DN. As podocytes are growth restricted, the loss of podocytes cannot be compensated, resulting finally in the development of ESRD. Therefore, it is of great interest to identify pathways that are responsible for the dedifferentiation process and for the detachment of podocytes.^4^, ^50^ Since several years, there is an ongoing debate whether podocyte progenitor cells exists in or nearby the glomerulus. Recently, Moeller and Romagnani have shown that PECs, flat epithelial cells covering the inner side of the Bowman`s capsule, could function as progenitor cells under specific circumstances.^8^, ^9^ Therefore, it is of great interest to reveal the mechanisms involved in a possible transformation of PECs into podocyte‐like cells or podocytes as it was described for other cell types like neurons and cardiomyocytes, whereby Parmar and colleagues showed that fibroblasts could directly differentiate into neurons after transfection with 3 specific transcription factors.^17^

Several publications have shown that the transcription and cell fate determination factor Dach1 is essential for the differentiation of embryonic cells into specific cell types.^21^, ^23^, ^25^, ^51^ Loss of *Dach1* in mice is lethal and the knockout in breast cancer cells, for instance, resulted in highly dynamic and proliferating cells.^25^ Furthermore, Dach1 was found to play an important role for renal function.^18^, ^19^, ^20^ However, almost nothing is known about the expression of *Dach1* in podocytes and its function in glomeruli. As a potential candidate for the transdifferentiation of PECs into podocyte‐like cells, we studied the effect of Dach1 in PECs that endogenously express only a very low amount of Dach1. We found that Dach1 expressing PECs significantly up‐regulate the podocyte‐specific protein synaptopodin, which is only faintly expressed in PECs. This is notable because synaptopodin is exclusively expressed in cell culture by well‐differentiated podocytes.^36^ Moreover, synaptopodin in Dach1‐expressing PECs was located along single actin filaments in a punctate pattern similar to that observed in differentiated podocytes, suggesting a transition of PECs into podocyte‐like cells. Furthermore, the expression of the podocyte‐specific transcription factor WT1, which is essential for the formation of foot processes and an intact filtration barrier,^52^, ^53^, ^54^ was significantly increased by Dach1 on the protein level.

As confirmed by RT‐PCR and high throughput RNA‐sequencing, Dach1 and synaptopodin mRNA levels were distinctly reduced in dedifferentiating podocytes of isolated glomeruli. Since other podocyte specific markers like Cd2ap and podoplanin were significantly up‐regulated after 9 days in culture, this might support the hypothesis that synaptopodin is Dach1‐dependently regulated in dedifferentiating podocytes.

To study the in vivo situation, we used the zebrafish as an animal model that is well established to study the role of specific proteins in a simplified kidney, the pronephros.^32^, ^33^, ^46^, ^55^ For instance, the Drummond's group has shown that the KD of nephrin and podocin causes foot process effacement resulting in leakage of the glomerular filtration barrier.^46^ As the knockout of Dach1 is lethal, we used the well‐established morpholino technique to KD Dachd, the ortholog of mouse and human *DACH1*, in zebrafish larvae. We have found that the KD larvae developed a severe glomerular phenotype associated with a reduction in the expression of nephrin indicating a dedifferentiation of podocytes.

Beside the typical podocyte‐specific proteins, we studied the regulation of Eya1, Eya3 and Pax2, transcription factors that are known to be involved in the Dach‐Eya‐Hox‐Pax cell fate network. Eya1 has already been shown to be essential for kidney development as Eya1^−/−^ mice develop only very small kidneys.^30^ In contrast to Pax2, a postulated PEC maker, which was down‐regulated by Dach1, the expression of Eya1 and Eya3 remained unaffected by the Dach1 expression in PECs, indicating that the Dach1 regulation is probably independent of this network. This has already been suggested by Brunskill et al.^56^

To find out whether the Dach1 expression is also altered in patients suffering from glomerular disease, we measured the *DACH1* mRNA expression of microdissected glomeruli and co‐stained biopsies for DACH1 and synaptopodin. In both experiments, we found a strong down‐regulation of DACH1 and a down‐regulation of synaptopodin in DN suggesting a correlation between both proteins. This hypothesis is reinforced by the result that the *DACH1* and synaptopodin mRNA expression of isolated mouse glomeruli were significantly down‐regulated after 9 days in cell culture in contrast to other proteins. Furthermore, our findings are in line with a recent publication reporting a positive correlation between glomerular DACH1 staining and the estimated glomerular filtration rate in patients suffering from glomerulopathy.^57^

Taken together, our results show that the transcription factor Dach1 is essential for proper podocyte function. Furthermore, Dach1 expression in PECs induces the expression of synaptopodin, which is expressed in differentiated podocytes. Therefore, Dach1 might be a novel important cell fate determination factor for podocytes.

## CONFLICT OF INTEREST

The authors confirm that there are no conflicts of interest.

## AUTHOR CONTRIBUTIONS

The study was designed by N.E. and K.E.; K.S., A.M.K. and R.M. contributed to the zebrafish experiments and Fe.K., F.D., R.M., N.K. and A.B. to the cell culture experiments; Fr.K. performed experiments on isolated glomeruli; biopsies were handled and analysed by N.A., M.T.L., C.D.C. and K.A.; RNA sequencing was performed by A.W.K; all other experiments were performed by N.E.; experimental data were analysed by N.E., M.J.M. and K.E.; N.E., Fe.K. and K.E. wrote the manuscript.
