# Supplementary material for: The transcription factor Dach1 is essential for podocyte function
Source: J Cell Mol Med. 2018 Mar 2;22(5):2656–69. doi: 10.1111/jcmm.13544 (PMC5908116; doi:10.1111/jcmm.13544)
Supplement: Supplementary file 1 [file JCMM-22-2656-s001.pdf]

Supplementary information

Supplementary Figures and Tables.

Supplementary Figure 1

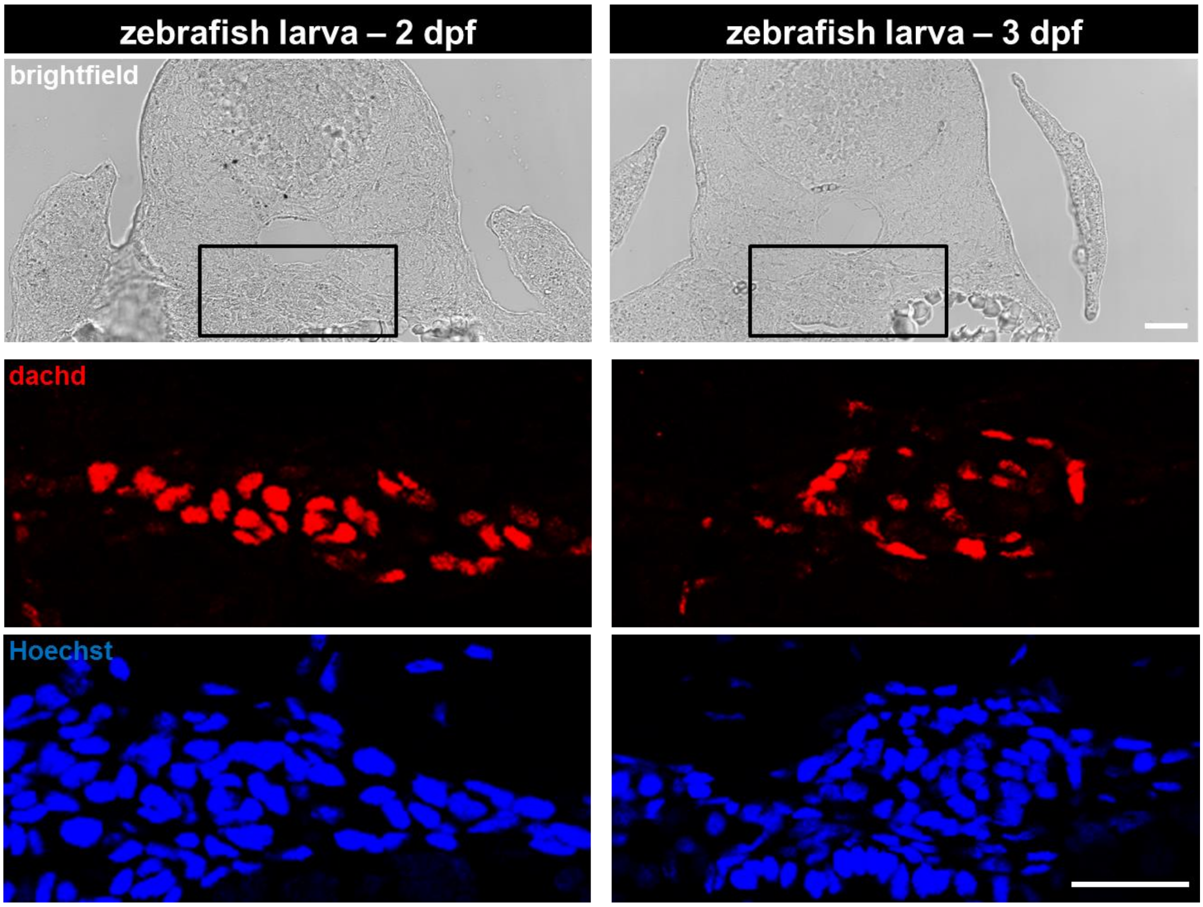

Supplementary Figure 1.

Dachd expression in glomeruli of 2 dpf and 3 dpf zebrafish larvae.

Paraffin sections of 2 dpf and 3 dpf zebrafish larvae showed a strong dachd expression (red, middle lane). Scale bar represents 20 µm.

Supplementary Figure 2

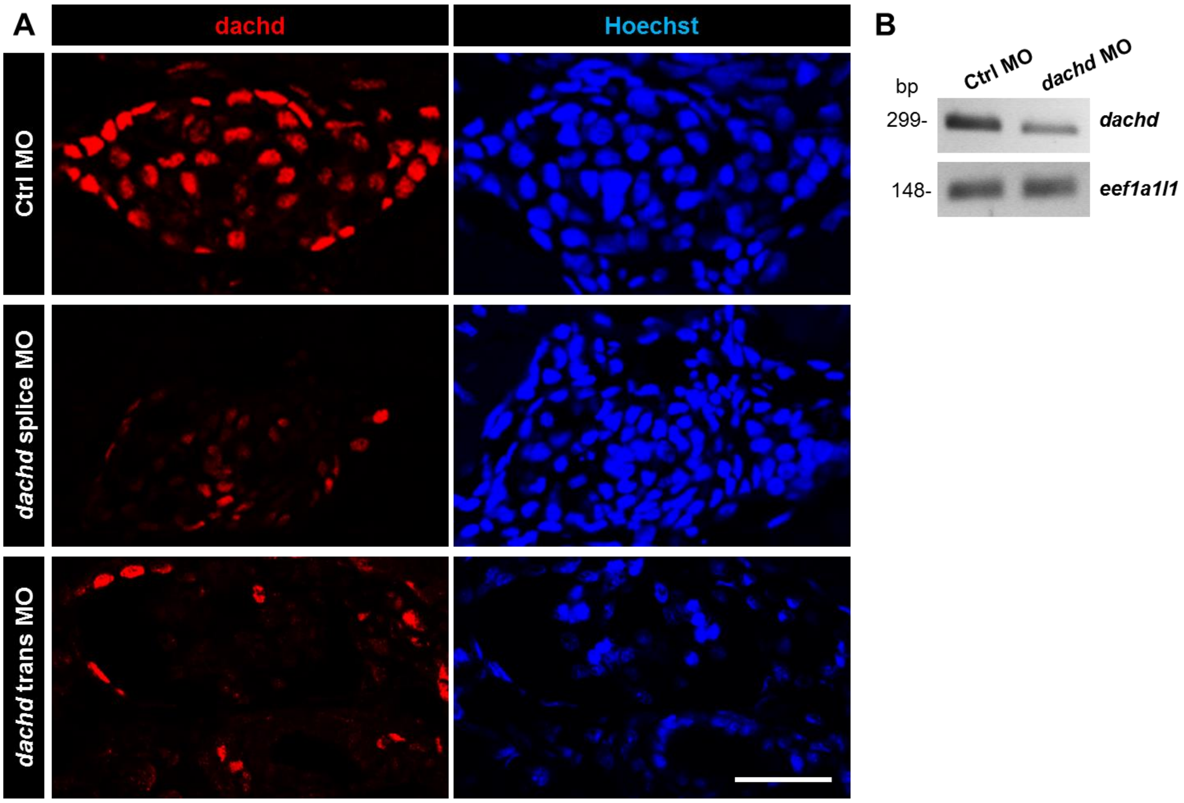

Supplementary Figure 2.

Confirmation of the *dachd* morpholino knockdown.

(A) Paraffin sections of 3 dpf zebrafish larvae showed a strong downregulation of the dachd expression (red) due to the injection of a *dachd* splice and dachd trans MO, respectively. Scale bar represents 20 µm. (B) The dachd mRNA downregulation was shown by RT-PCR analysis. Total RNA of larvae (3 dpf) that were injected with Ctrl MO and *dachd* splice MO.

Supplemental Table 1: qRT-PCR Primer that were used for mouse[1]/zebrafish[2] .

Detected gene	Sequence
Gapdh[1]	F: GGGTTCCTATAAATACGGACTGC<br>R: CCATTTTGTCTACGGGACGA
Actin[1]	F: CACCCGCGAGCACAGCTTCT<br>R: CCACCATCACACCCTGGTGCC
18sRNA[1]	F: GTAACCCGTTGAACCCCATT<br>R: CCATCCAATCGGTAGTAGCG
Dach1[1]	F: GCAAACTCATCTCCAGGAAGG<br>R: GCATGATGTGAGAGTTCTCTGG
Synpo[1]	F: TCCCAGGCCTTCCTTCTC<br>R: AGGGGGACATTGGTGGAG
Wt1[1]	F: CCGACCATCTGAAGACCCAC<br>R: ACACTTTCCTGCCTGGGATG
Nphs2[1]	F: CACCGTAGTGGACGTGGAC<br>R: GCAGGCCCCTAATCCAGAG
Pax2[1]	F: GGCTGTGTCAGCAAAATCCTG<br>R: GTCGGGTTCTGTCGCTTGTATTC
Cav1[1]	F: GATTGACCTGGTCAACCGC<br>R: CTTCCAGATGCCGTCGAAAC
Eya3[1]	F: CCAGACGGAGAAGCCTAGTG<br>R: CTGGCCTGATCATCAGCATC
Eya1[1]	F: CCACTTACCAACTCCAGGAACC<br>R: CATCTGAACCTCGACGCAG
zEef1a1l1[2]	F: AAGGAGGGTAATGCTAGCGG<br>R: GGGCGAAGGTCACAACCATA
zSynpo[2]	F: CGCCTCAAACTGCAGGATC<br>R: CCTGCTTAATCTGTGGGGC
zNephrin[2]	F: CAATGTCCCTAACCCGCACT<br>R: ACGCCTCACATTGCAGAGAA
zPodocin[2]	F: GGCCCTGGGCTGATGTTTTA<br>R: GAGCAATGCGTTTCCTGTCC
zRpl13α[2]	F: TCTGGAGGACTGTAAGAGGTATGC<br>R: AGACGCACAATCTTGAGAGCAG
z18sRNA[2]	F: TTACCCCAGGCTCGGAAAAC<br>R: CGGGAAGGTCTTTGAACCCA

*zdachd*_trans	F: CAGACAGAACACACCATGGC<br>R: GTCTTGAGCTGGCATTGGTG
*zdachd*_splice	F: GTGTAACGTGGAGCAGGTC<br>R: GTCCGTTTTGGAGGCCTC
zdachd_Exon1/3	F: CCTCTACAACGACTGCACCA<br>R: TGGAGCTCAGGTCTCCATTC

Supplemental table 2: Sequence of the zebrafish morpholinos that were used for injection into fertilized eggs.

**Morpholino**	**Sequence**	**Blocking type**
dachd splice	tgagctaaaacagaagtgaagtggt	Splice blocking (1e2)
dachd trans	catggtgtgttctgtctgcctgtcc	Trans blocking
dachd vivo	catggtgtgttctgtctgcctgtcc	*in vivo* trans blocking
control MO	cctcttacctcagttacaatttata	negative control
control *vivo* MO	cctcttacctcagttacaatttata	negative control *vivo*
